# Monoclonal immunoglobulin mediates complement activation in monoclonal gammopathy associated-C3 glomerulonephritis

**DOI:** 10.1186/s12882-019-1640-3

**Published:** 2019-12-10

**Authors:** Lin-Lin Li, Zhi-Ying Li, Su-Xia Wang, Xiao-Juan Yu, Ying Tan, Yu Wang, Feng Yu, Ming-Hui Zhao

**Affiliations:** 1 Renal Division, Department of Medicine, Peking University First Hospital, Institute of Nephrology, Peking University, Beijing, 100034 People’s Republic of China; 2 Key Laboratory of Renal Disease, Ministry of Health of China, Key Laboratory of CKD Prevention and Treatment, Ministry of Education of China, Beijing, 100034 People’s Republic of China; 3 Laboratory of Electron Microscopy, Pathological Centre, Peking University First Hospital, Beijing, 100034 People’s Republic of China; 4 Department of Nephrology, Peking University International Hospital, Beijing, 102206 People’s Republic of China; 5 Peking-Tsinghua Center for Life Sciences, Beijing, People’s Republic of China

**Keywords:** C3 glomerulonephritis, Anti-CFH autoantibodies, Monoclonal immunoglobulin (MIg), Monoclonal gammopathy of renal significance (MGRS)

## Abstract

**Background:**

C3 glomerulonephritis (C3GN) is a rare disease caused by inherited or acquired complement alternative pathway (CAP) dysregulation, which could also be secondary to monoclonal gammopathy of undetermined significance (MGUS). Herein, we described a patient presenting with C3GN and monoclonal gammopathy, and the pathogenic association between the two diseases was further explored in vitro.

**Case presentation:**

A 76-year-old Chinese man presented with low serum C3 level, haematuria and nephrotic syndrome, and experienced rapid worsening of renal function over a period of 10 months. His serum and urine immunofixation electrophoresis both revealed a monoclonal IgGλ. A bone marrow puncture showed plasma cell dyscrasias with the highest plasma cell count of 5.25%. Kidney biopsy showed the presence of C3 glomerulonephritis, with exclusive deposits of C3 visible on immunofluorescence, a membranoproliferative pattern on light microscopy and electron dense deposits in sub-epithelial, intramembranous, sub-endothelial and mesangial regions by electron microscopy. The patient was positive for C3 nephritic factor (C3NeF) activity and anti-CFH autoantibodies, and all became negative during disease remission. The anti-CFH autoantibodies purified from the patient’s plasma exchange fluids were proven to be a monoclonal IgGλ, and could inhibit CFH binding to C3b and accelerate the formation of C3 convertase indirectly by interfering with the formation-impeding activity of CFH. No deficiency of candidate genes, especially variants in CFH, was detected in our patient. Based on the pathological and laboratory findings, the diagnosis of monoclonal gammopathy of renal significance (MGRS)-associated C3GN was finally made.

**Conclusions:**

This is the first demonstration that intact monoclonal immunoglobulin (IgGλ) could act as an anti-CFH antibody and lead to MGRS-associated C3GN by activating the CAP.

## Background

C3 glomerulopathy (C3G) is characterized by predominant glomerular C3 fragment deposition with electron-dense deposits on electron microscopy. The disease is thought to be caused by excessive activation of the complement alternative pathway (CAP) and serum C3 levels are usually low. According to the distribution of electron-dense deposits on electron microscopy, C3G could be subdivided into dense deposit disease (DDD) and C3 glomerulonephritis (C3GN) [1, 2]. C3G results from acquired or genetic abnormalities in the CAP, such as the presence of C3 nephritic factor (C3NeF), antibodies or gene variants/mutations for complement factor H (CFH) or complement factor B (CFB), etc. [3, 4]. Monoclonal immunoglobulins (MIg) may also play a causal role in C3G by impairing the regulation of the CAP [5]. The terminology MGRS (monoclonal gammopathy of renal significance) is used to denote a monoclonal gammopathy of undetermined significance that is responsible for a renal disease [6, 7]. Recently, a link between C3G, monoclonal gammopathy and MGRS has been observed, especially in older adults [8–15], although the role of MIg in the pathogenesis of C3G remains to be elucidated. We herein described a patient presenting with C3GN and monoclonal gammopathy, and the pathogenic association between the two diseases was further explored in vitro.

## Case presentation

### Case descriptions

A 76-year-old Chinese man presented with microscopic haematuria for 2 years and oedema for 8 months. The patient had a past history of age-related macular degeneration (AMD), hypertension, angina pectoris and hypothyroidism. On admission, the physical examination revealed a blood pressure of 145/76 mmHg, a temperature of 36.5 °C, a heart rate of 76 beats/min, and a respiratory rate of 22 breaths/min. The patient had severe bilateral symmetrical lower extremity oedema. Urine dipstick showed blood (3+) and protein (3+), and urinalysis showed with 80–90 RBCs/HPF with a majority of dysmorphic RBCs. Laboratory findings included a serum albumin concentration of 20 g/L and a proteinuria value of 8.06 g/d. His serum creatinine value rose from 1.41 mg/dL to 2.96 mg/dL in 10 months and decreased to 1.81 mg/dL after diuretic therapy. His haemoglobin level was 77 g/L (normal range: 130–175 g/L) and his platelet count was 212 × 109 cells/L (normal range: 125–350 × 109 cells/L). His C3 level was low at 0.356 g/L (normal range: 0.6–1.5 g/L), his C4 level was normal at 0.162 g/L (normal range: 0.12–0.36 g/L) and his plasma CFH level was normal at 392.9 μg/mL (normal range: 247–1010.8 μg/mL). His serum IgG level was 9.89 g/L (normal range: 7.23–16.85 g/L), his IgA level was 2.38 g/L (normal range: 0.69–3.82 g/L), and his IgM level was 0.78 g/L (normal range: 0.63–2.77 g/L). His serum and urine immunofixation electrophoresis both revealed a monoclonal IgGλ. A bone marrow puncture was performed, and the diagnosis of plasma cell dyscrasias was made, with the highest plasma cell count of 5.25%.

**Fig. 1:**
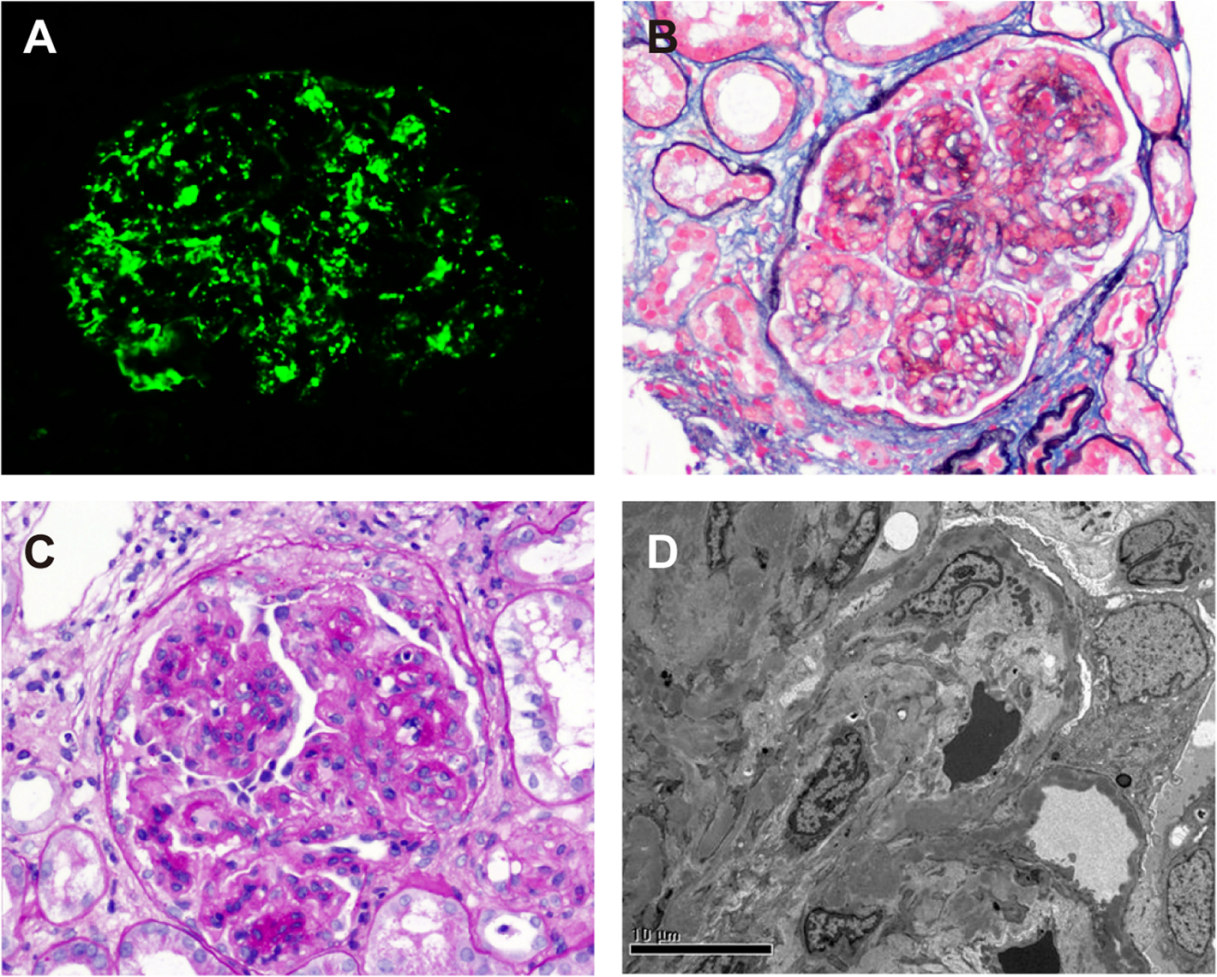
Representative figures of renal biopsy. **a** Intensive granular deposits of C3 (3 + −4+) were detected in the mesangial regions and segmental deposits along the capillary walls (immunofluorescence staining on frozen tissue, × 400). **b** Severe mesangial proliferation and interposition to form a lobular appearance, along with a thickening of the glomerular capillary wall with double contours (periodic acid-silver methenamine + Masson trichrome staining, × 400). **c** Severe mesangial proliferation with endocapillary hypercellularity and a small fibro-cellular crescent (Periodic acid staining, × 400). **d** Electron dense deposits in sub-endothelia, intramembranous and mesangial regions viewed by electron microscopy (× 8000)

The patient underwent a renal biopsy 2 days after hospitalization. By immunofluorescence, intensive granular deposits of C3 (3 + − 4+) were detected in the mesangial regions and segmental deposits along the capillary walls (Fig. 1a). C1q was trace, and no deposits of IgG, IgA, IgM, or κ and λ light chains were detected. By light microscopy, there were 12 glomeruli in the specimen, and the appearance of glomerular lesions was characterized by severe mesangial proliferation and interposition and endocapillary hypercellularity to form lobular and thickened glomerular basement membranes (GBMs) with double contours. Fuchsinophilic deposits were identified in the sub-endothelial and mesangial regions. Two of 12 glomeruli showed small fibro-cellular crescents. Degenerative changes in tubular epithelia were mild and a focal interstitial infiltration of lymphocytes with fibrosis was identified. Arteriolar sclerosis was mild (Fig. 1b and c). Electron microscopy revealed moderate to severe mesangial proliferation with interposition and a thickening of the GBM with a segmental widening of sub-endothelia regions was found. Electron-dense deposits were present in the sub-epithelial, intramembranous, sub-endothelial and mesangial regions (Fig. 1d). A severe foot process effacement of podocytes was identified. A final diagnosis of C3 glomerulonephritis with a pattern of membranoproliferative glomerulonephritis (MPGN) was then made.

### Detection of plasma C3NeF activity and anti-CFH or CFH SCR autoantibodies

The patient was positive for C3NeF activity. His anti-full length CFH and CFH SCR7 autoantibodies were also found to be positive by ELISA, and the subclass of anti-full-length CFH autoantibodies was IgG3.

**Fig. 2:**
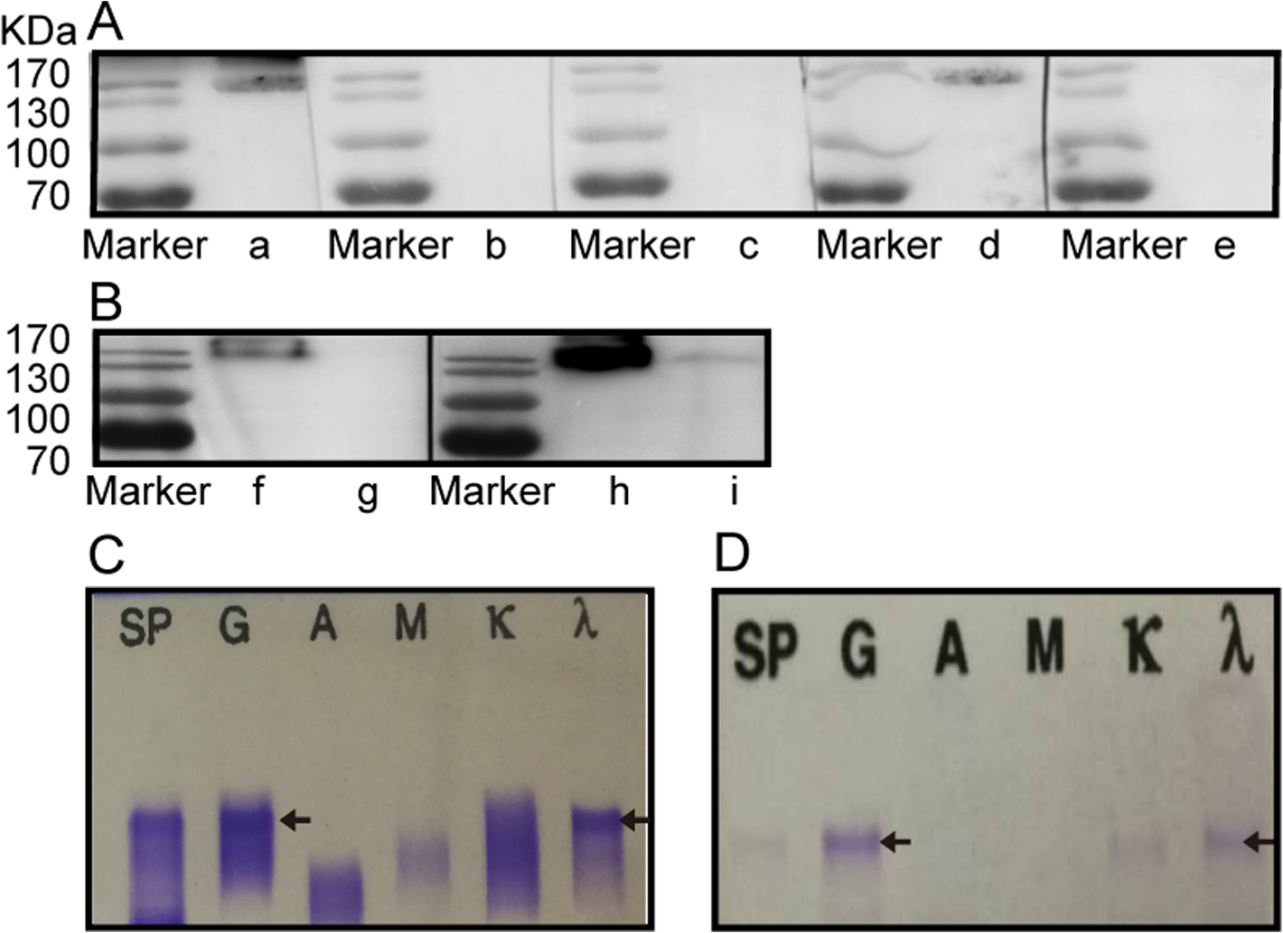
The purified anti-CFH autoantibody. **a** Confirmation of purified anti-CFH autoantibodies from our patient by Western blot. Commercial human CFH (1 μg) under non-reducing conditions was electrophoresed on 10% SDS-PAGE and transferred to a PVDF paper, blocked and then incubated with anti-CFH autoantibodies purified from our patient. The anti-CFH autoantibodies purified from our patient, which was IgG3λ, could recognize commercial CFH. Lanes a-e were incubated with the followings antibodies: anti-human IgG, IgG1, IgG2, IgG3, and IgG4, respectively. **b** The anti-CFH autoantibodies purified from our patient had lambda light chain without a kappa chain. Lanes f and h, lanes g and i were loaded with anti-CFH autoantibodies purified from the aHUS patient and our patient, respectively, under non-reducing conditions on 8% SDS-PAGE; lanes f and g were then incubated with anti-human kappa chain, and lanes h and i were incubated with anti-human lambda chain. **c** The serum of our patient contained a monoclonal IgGλ by immunofixation electrophoresis assay. **d** The purified anti-CFH autoantibodies from our patient were confirmed to be the same monoclonal IgGλ by immunofixation electrophoresis assay

His anti-CFH autoantibodies were purified from plasma exchange fluids using Protein G and CFH affinity column, and were proven to be a monoclonal IgGλ by both Western blot and immunofixation electrophoresis assays, which was consistent with the serum monoclonal IgGλ (Fig. 2a-d).

### Bio-functional analyses of anti-CFH autoantibodies in vitro

#### C3b binding assay

**Fig. 3:**
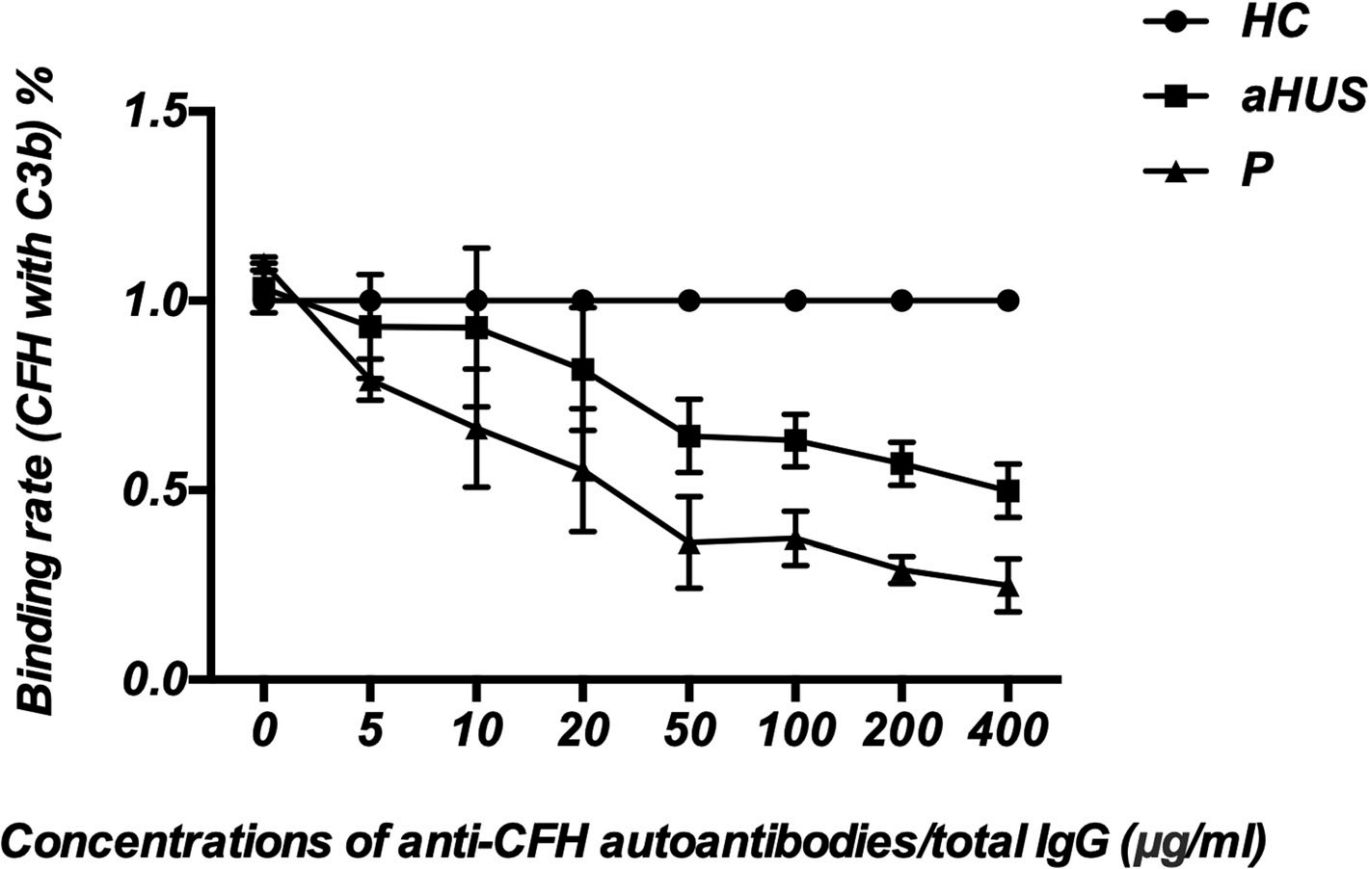
Binding assay of CFH to C3b. Inhibition of the interaction between CFH and C3b as measured by ELISA. The anti-CFH autoantibodies purified from our patient and the aHUS patient, and the total IgG from three healthy controls were analysed individually. The percentage of inhibition was calculated by defining the average optical density (OD) value obtained in the presence of each healthy control IgG to be 1. The purified anti-CFH autoantibodies from our patient inhibited the interaction between CFH and C3b in a dose-dependent manner. Each assay was performed three times, and the data are shown as the mean ± SD. Notes: P: Our patient; aHUS: the aHUS patient with anti-CFH autoantibodies; HC: healthy controls

As shown in Fig. 3, the binding of CFH to C3b was reduced significantly in a dose-dependent manner with autoantibodies from our patient compared with IgG from 3 healthy controls (*P* < 0.001), which was even worse than the level interfered by the anti-CFH autoantibodies from an aHUS patient (*P* = 0.001).

#### C3 convertase formation and decay assay

**Fig. 4:**
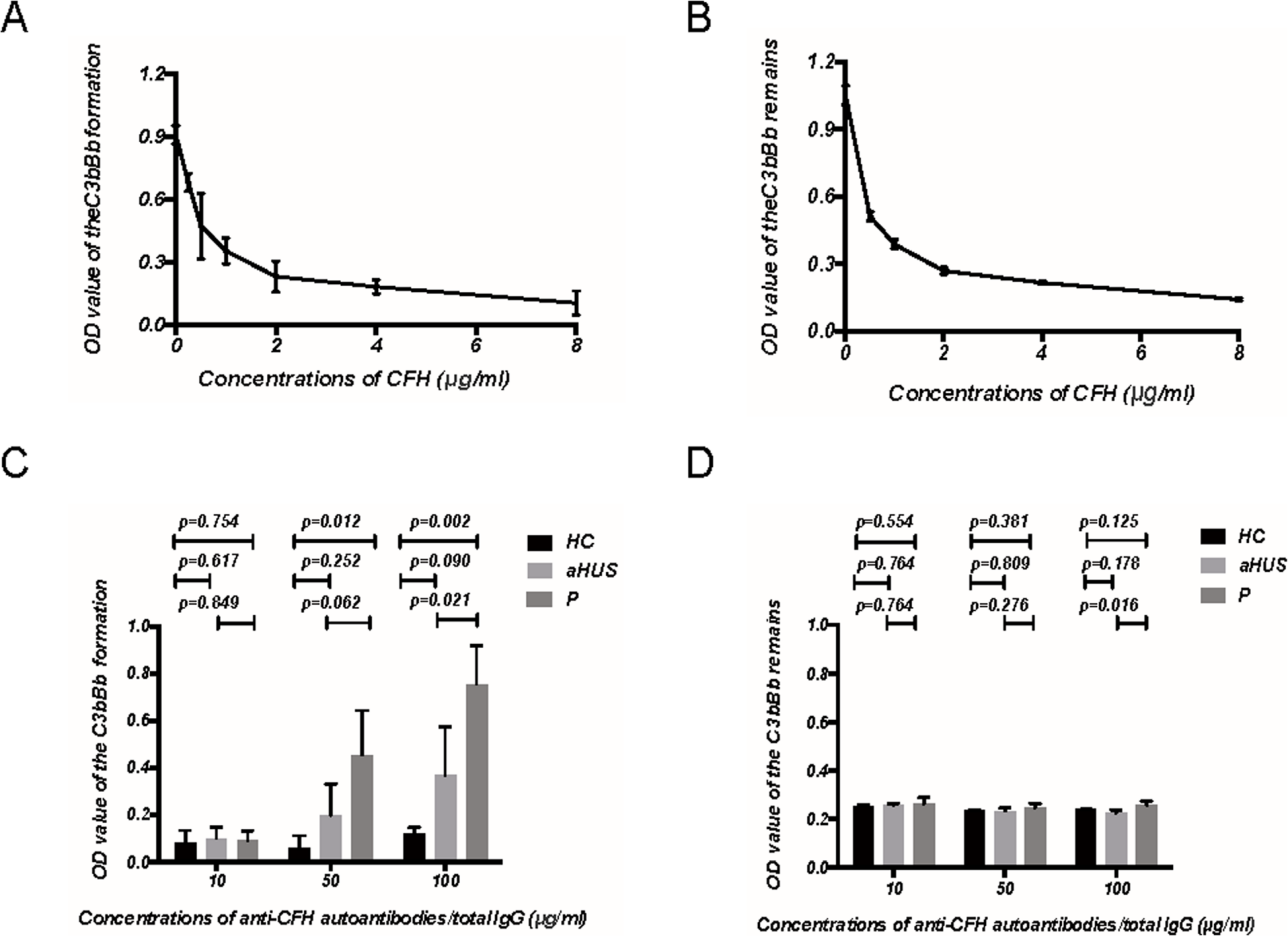
C3 convertase formation assay and decay assay. **a** Commercial human CFH exhibits a strong formation-impeding activity of the C3 convertase (C3bBb) assembled on immobilized C3b in a dose-dependent manner. **b** Commercial human CFH accelerates the decay of the C3 convertase (C3bBb) assembled on immobilized C3b in a dose-dependent manner. **c**, **d** Pre-incubated CFH (4 μg/ml) with purified anti-CFH autoantibodies from our patient and the aHUS patient, and total IgG from three healthy control individually at concentrations of 10 μg/ml, 50 μg/ml, and 100 μg/ml, respectively. Anti-CFH autoantibodies purified from our patient showed significantly reduced formation-impeding activities in a dose-dependent manner (**c**) and no significant effects on the decay-accelerating activities of CFH (**d**). Notes: P: Our patient; aHUS: the aHUS patient with anti-CFH autoantibodies; HC: healthy controls. Each assay was performed three times, and the data are shown as the mean ± SD

We tested the capacity of CFH to interfere with the formation and accelerate the decay of the alternative pathway C3 convertase (C3bBb), which was assembled on immobilized C3b. Commercial CFH exhibited strong formation-impeding and decay-accelerating activity in a dose-dependent manner (Fig. 4a and b). Auto-antibodies against CFH purified from our patient showed significantly reduced formation-impeding activities in a dose-dependent manner compared with IgG from 3 healthy controls and the aHUS patient (Fig. 4c). There was no significant difference in the decay-accelerating activities between any purified antibodies with commercial CFH and commercial CFH only for the C3 convertase decay (Fig. 4d).

### Genotyping

An integrated screening of 34 genes from the complement system using targeted genomic enrichment and massively parallel sequencing was performed, including C1QA, C1QB, C1QC, C1R, C1S, C2, C3, C5, C6, C7, C8A, C8G and C9; Plasma protease C1 inhibitor (SERPING1); Complement factor B, D, P, I and H; Complement factor H-related protein 1–5; Thrombomodulin (THBD); Mannose-binding lectin serine protease 1 and 2 (MASP1, MASP2); Collectin-11 (COLEC11); Complement receptor type 2 (CR2); Integrin beta-29 (ITGB2); Membrane cofactor protein 46 (CD46); Membrane cofactor protein 59 (CD59); and Ficolin-3 (FCN3). No deficiency of a single gene or candidate genes, especially variants in CFH, was detected in our patient.

### Final diagnosis, treatment and follow-up

**Fig. 5:**
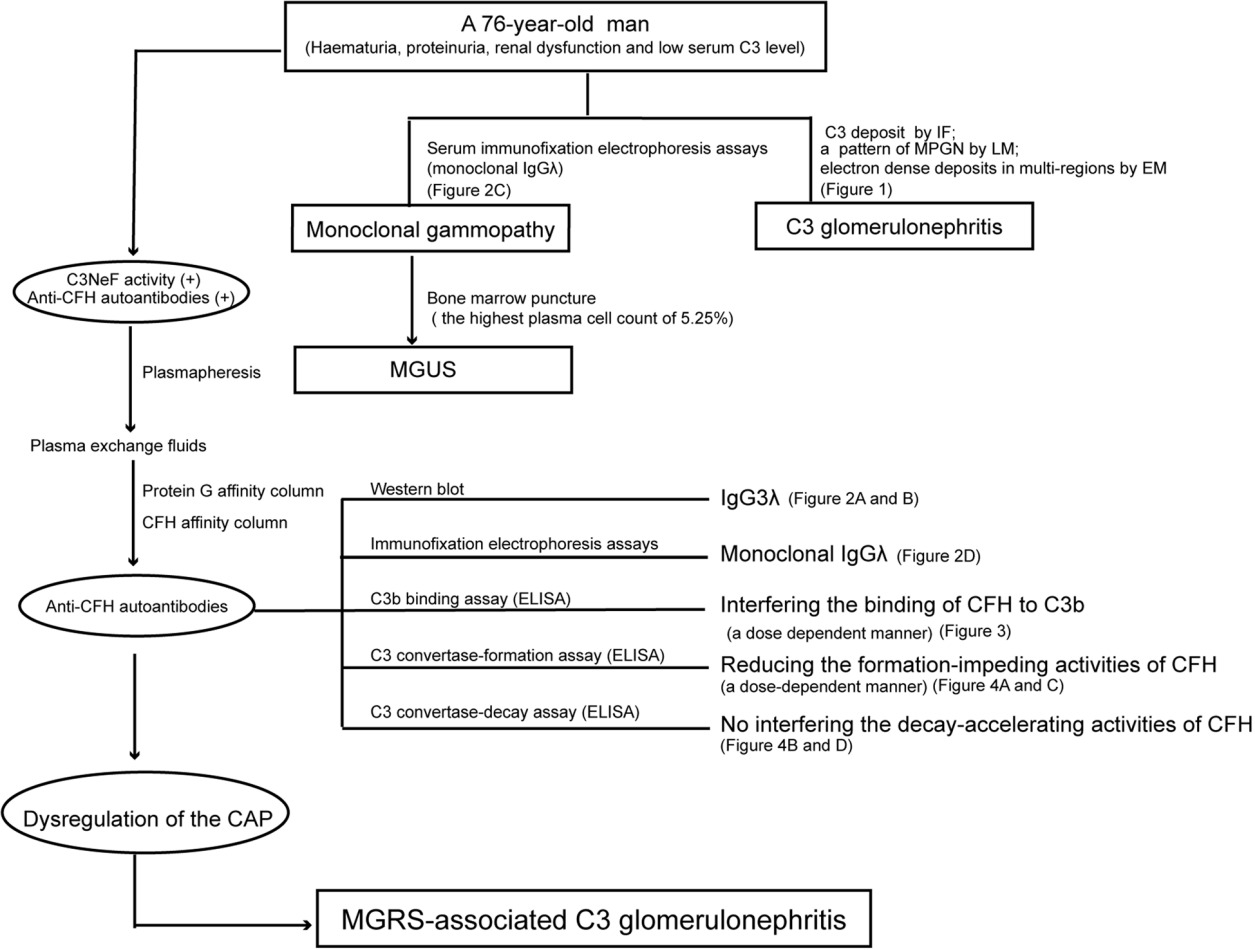
Flow chart of our patient. Notes: IF: Immunofluorescence microscopy; LM: Light microscopy; EM: Electron microscopy; MGUS: Monoclonal gammopathy of undetermined significance; MGRS: Monoclonal gammopathy of renal significance; C3NeF: C3 Nephritic factor; CAP: Alternative pathway of complement; ELISA: enzyme-linked immunosorbent assay

Based on the pathological and laboratory findings, the diagnosis of C3GN in our patient, which might be due to MGRS, was finally made (Detailed flow chart in Fig. 5). During the exploration of the underlying causes for C3GN in this patient, we found that the patient was positive for C3NeF activity and anti-CFH autoantibodies. His anti-CFH autoantibodies were proven to be a monoclonal IgGλ, which corresponded to his serum and urine immunofixation electrophoresis results.

Six rounds of plasmapheresis were initiated, and the chemotherapy with bortezomib was then recommended although the patient declined the latter therapy. He received oral prednisone (initial daily dose of 1 mg/kg, tapered slowly) and cyclophosphamide (daily dose of 50 mg) with an accumulate doses of 4.5 g in combination with a calcium channel blocker and erythropoiesis-stimulating agent therapy. Ten months later, his proteinuria amount had decreased to 0.24 g/d, his serum albumin level had increased to 40 g/L, his serum creatinine value was stable (1.39–1.46 mg/dL), his serum C3 level had increased to 0.92 g/L, his plasma CFH level had increased to 833 μg/ml, and his plasma autoantibodies against CFH and plasma C3NeF activity had both turned negative. His serum monoclonal λ light chains were trace, and neither monoclonal IgGλ nor monoclonal λ light chains could be detected in his urine.

## Discussion and conclusions

In this study, the definitive diagnosis of C3G was made based on the demonstration of C3 deposits by immunofluorescence and membranoproliferative glomerulonephritis (MPGN) and electron dense deposits in sub-epithelial, intramembranous, sub-endothelial and mesangial regions by electron microscopy [7] and monoclonal gammopathy was shown by the demonstration of a serum monoclonal IgGλ by immunofixation electrophoresis assays. The associations between C3G and monoclonal gammopathy were further explored in vitro.

The mechanisms that contribute to C3G are multiple, including the presence of C3NeF, antibodies and/or gene variants/mutations of complement-regulating proteins, etc. [3, 4]. Recently, more attention was paid to the link between C3G and monoclonal gammopathy, especially in older adults [5, 8–10, 12, 13, 16, 17], as 65.1% of C3G patients with 50 years of age and older had a monoclonal gammopathy [16], which is nearly 16 times higher than the prevalence (3.2%) of monoclonal gammopathy in the general population [18].

We reviewed and summarized published C3G combined monoclonal gammopathy cases in the Table 1 [5, 8–10, 12–17, 19–25]. In brief, C3G with monoclonal gammopathy mainly concerned older people, with a striking male predominance. IgG κ was the most common MIg isotype, and the vast majority of the patients were classified as MGRS/MGUS. In renal ultrastructural pattern, C3GN was more common than DDD, although there was an overlap of cohort between Chauvet’s two series studies. The renal outcomes of C3G was poor in most cases, and the improvement of renal parameters after chemotherapy in a few patients suggested that MIg might have a pathogenic role in CAP activation. In some cases, C3NeF, anti-CFH autoantibodies, and anti-CR1 autoantibodies could be detected, and it was assumed that MIg could act as an antibody to complement fragments such as C3 convertase or CFH, interfering with bio-functions and resulting in the over-activity of the CAP [8, 9], although the precise mechanism remained to be elucidated. Importantly, we found that the subclass of anti-CFH autoantibodies in our patient was IgG3, which is poorly represented in normal polyclonal IgG. IgG is divided into four subclasses, named, in order of decreasing abundance IgG1, IgG2, IgG3 and IgG4, which are highly homologous and differ in their constant region, particularly in their hinges and upper CH2 domains [26]. These regions are involved in binding to both IgG-Fc receptors (FcγR) and C1q. IgG3 has a much longer region than any of the other IgG subclasses, which results in its higher molecular mass and the difference in hinge flexibility [27]. The difference in flexibility influences the binding of IgG3 to both C1q and FcγR, and affects the antigen-binding capacity and immune complex formation, which results in a potent pro-inflammation and a particularly effective antibody [26]. The current work aimed to clarify the causal relationship between the MIg and the C3G occurrence, and further explorations of its IgG3 subclass are needed.

**Table 1:** Reports of C3G patients combined with monoclonal gammopathy in the literature

Authors	Year	Patients, n	Age	M/F, n/n	Serum MIg, n	Haematological diagnosis, n	Evaluation of factors affecting CAP, n	Electronic microscopy findings of renal biopsy, n	Treatment	Renal outcomes, n
Meri [19]	1992	1	57	0/1	λ LC: 1	MGRS	λ LC dimer could act as a mini-autoantibody against CFH	Subendothelial and intramembranous dense deposits in the GBM (consistent with DDD)	NA	NA
Jokiranta [20]	1999									
Sethi [9]	2010	10	60 (49–77)	2/8	IgGκ: 6 IgGλ: 3 IgA & IgGλ: 1	No patient had progressed beyond MGUS	FHAA and CFH H402 allele: 11	DDD: 10	No specific therapy	ESRD: 4; chronic kidney failure: 2; recent diagnosis: 4
Bridoux [8]	2011	6	67.5 (40–74)	3/3	IgGκ: 4	MGUS: 5	FHAA: 1	EM (performed in 5 patients): nonextensiveamorphous electron-dense deposits, or deposits of intermediate density, with a “sausage-shaped” appearance, were observed within the lamina densa (consistent with DDD)	No treatment: 2; Dex: 1; Mel+ Dex: 1; CYC + Dex: 1; Bortezomib+Dex: 1	ESRD: 5 (despite chemotherapy in 4 patients); chronic kidney failure: 1
					IgGλ: 2	Smouldering MM: 1	CFH H402 allele: 2			
Zand [10]	2013	10	61.5 (22–69)	7/3	IgGκ: 6	MGUS:9 CLL: 1	C3NeF: 2	C3GN: 10	Conservative: 4; Rituximab+CYC + vincristine+prednisone: 1; Dex + bortezomib: 1; immunosuppressive therapy: 4	ESRD: 3; relatively stable renal function: 6; improved renal funcion: 1
					IgGλ: 2		CFH H402 allele: 3			
					IgAλ: 1					
					IgMλ: 1					
Lloyd [5]	2016	10	63.5 (52–90)	9/3	IgGκ: 8	MGRS: 5	NA	DDD: 3; C3GN: 7	Chemotherapy (including bortezomib+DEX): 4 patients with MM	ESRD: 5 (three patients with MM); relatively stable renal function: 3; improved renal funcion: 2 (one patient underwent SCT)
					IgGλ: 1	MM: 4				
					IgAκ: 1	Polyclonal plasmacytosis: 1				
Yin [17]	2016	1	64	0/1	IgGλ: 1	MM	NA	C3GN	Thalidomide+DEX	recent diagnosis
Daccueil [14]	2017	1	61	1/0	IgGλ: 1	MGRS	NA	DDD	Rituximab+steroids	Improved renal function.
Hamzi [21]	2017	1	32	1/0	λ LC: 1	MM	Not done	Electronic microscopy was not performed	Chemotherapy (CYC + DEX + thalidomide) → SCT	Improved renal function.
Chauvet [12]	2017	50	65 (38–82)	33/17	IgGκ: 36	MGRS: 30	C3NeF: 3;	Electron microscopy was available in 25 cases. DDD: 1; C3GN: 24	Conservative: 13; immunosuppressive therapy: 8; clone-directed chemotherapy: 29 (including bortezomib in 22 patients)	ESRD: 25 (9 in the chemotherapy group)
					IgGλ: 11	Smouldering MM: 15	FHAA: 9			
					IgAκ: 1	Symptomatic MM: 2	a rare variant of undetermined significance (p. D130N in CFH and p. E548Q in CFI): 2			
					IgAλ: 1	CLL: 3				
					λ LC only: 1					
Timmermans [15]	2018	2	75.5 (75–76)	1/1	IgGκ: 1	MGUS: 2	C3NeF and FHAA were not found	DDD: 1; C3GN: 1	Conservative: 2	Relatively stable renal function: 1; chronic kidney failure: 1
					IgGλ: 1					
Chauvet [13]	2018	41	63 (36–83)	25/16	IgGκ: 27	MGRS: 28	C3NeF: 3;	Electron microscopy was available in 25 cases. DDD: 1; C3GN: 24	NA	NA
					IgGλ: 11	other (MM, CLL): 13	FHAA: 9;			
					IgAκ: 2		FIAA: 2;			
					κ LC only: 1		Anti-CR1 Abs: 11			
							a rare variant of undetermined significance (p. D130N in CFH and p. E548Q in CFI): 2			
Ravindran [11]	2018	36	60 (20–85)	25/11	IgG: 31	MGRS: 26	C3NeF: 11/24;	DDD: 4; C3GN: 32	Conservative: 3; non-targeted therapy: 17; MIg-targeted therapy: 16	Complete or partial renal response or stable renal function: 44% of patient with MIg-targeted therapy and 41% of patients with non-targeted therapy.
					IgM: 3	Smouldering MM: 2	FHAA: 2/24;			
					IgA: 1	Symptomatic MM: 5	FBAA: 1/24;			
					κ: 26	CLL: 1	CFH H402 and/or V62 allele: 13/21			
					λ: 10	Cryoglobulinemia (type 1): 1	other variants/mutations of unknown C3G pathogenicity: 4/212			
						Cryoglobulinemia (type 2): 1				
Hirashio [22]	2018	1	52	1/0	λ LC: 1	MGRS	FHAA	DDD	Bortezomib+DEX	Improved renal function and histological resolution of DDD.
Ramirez [23]	2018	1	70	1/0	IgGκ: 1	MGUS	Not done	NA	Intravenous methylprednisolone→PEX → MMF	Improved renal function
Lepori [24]	2018	1	55	1/0	IgGκ: 1	MGRS	Not done	C3GN	Mel→SCT	Relatively stable renal function
Moog [25]	2018	1	59	1/0	IgGλ: 1	Smouldering MM	Not done	DDD	Eculizumab, chemotherapy (bortezomib+DEX), PEX, immunosuppressive therapy	Relatively stable renal function

*Abbreviations*: *C3G* C3 glomerulopathy, *C3NeF* C3 nephritic factor, *CAP* Complement alternative pathway, *CFH* Complement factor H, *CLL* Chronic lymphocytic leukaemia, *FHAA* Factor H autoantibody, *LC* Light chain, *MGRS* Monoclonal gammopathy of renal significance, *MGUS* Monoclonal gammopathy of undetermined significance, *MIg* Monoclonal immunoglobulin, *MM* Multiple myeloma, *MPGN* Membranoproliferative glomerulonephritis, *ESRD* End-stage renal disease, *SCT* Stem cell transplantation, *Dex* High-dose dexamethasone, *Mel* Melphalan, *CYC* Cyclophosphamide, *PEX* Plasma exchange, *MMF* Mofetil mycophenolate, *NA* Not available

^1^Evaluation was not performed in the remaining 9 patients

^2^Variants/mutations of unknown C3G pathogenicity, including *APCS*, *C1QA*, *F5*, *DGK*, *FCN1*, and *PLG*

In our patient, anti-CFH autoantibody and MIg (IgGλ) were both demonstrated in the serum. In further explorations, we purified the intact and specific IgG against CFH directly and found that the purified antibody was a monoclonal IgGλ, which could inhibit the CFH binding to C3b in a dose-dependent manner and accelerate the formation of C3 convertase (C3bBb) indirectly by interfering with the formation-impeding activity of CFH. Our results highlighted that the MIg-C3G could be attributed to the over-activation of the CAP by the monoclonal anti-CFH IgGλ. In a previous study, Meri et al. reported that the Ig λ-chain dimer purified from a patient with membranoproliferative glomerulonephritis served as a mini-antibody directed against CFH SCR3 and was responsible for CAP activation before C3GN was described as a separate entity [19, 20], which is consistent with our findings concerning the monoclonal IgGλ of our patient. Importantly, more direct evidences focusing on the effects of the dysregulations of CFH on the C3 convertase, could better reflect the uncontrolled CAP activation from our patient.

Interestingly, the C3NeF activity was also positive in our patient and it turned negative with the disappearance of anti-CFH autoantibodies during disease remission, although the anti-CFH autoantibodies failed to stabilize the C3 convertase directly in our in vitro experiments. It is suggested that the C3NeF, a group of autoantibodies detected in the majority of DDD (86%) and less (45%) in C3GN patients [4], could bind to neo-epitopes in the newly assembled C3bBb and increase the half-life of the convertase by stabilizing it against both intrinsic and extrinsic CFH-mediated decay [28, 29]. However, the standard methods of measuring C3NeF are not currently well established: it is usually identified by residual Bb, haemolysis assays or C3 breakdown products, and rarely by the direct detection of autoantibodies [28]. We used the C3NeF stabilization ELISA with properdin (COS-P) to identify C3NeF indirectly here. With further explorations, we found that the anti-CFH autoantibodies could inhibit the CFH binding to C3b and interfered with formation-impeding activity of CFH, thus directly causing the stabilization of C3 convertase. Thus, we hypothesized that the anti-CFH autoantibodies were distinct from the classical C3NeF, and the detected C3NeF activity in our study might be due to the effects of the dysregulation of CFH on the C3 convertase.

In this case, our patient was successfully treated using immunosuppressive therapy with oral prednisone plus cyclophosphamide although he denied the bortezomib. The treatment for C3G patients with monoclonal gammopathy are mainly based on clinical opinion and experience by now, as there lacked confirmed guidance. In 2013, the study from the Mayo Clinic suggested that if monoclonal gammopathy was due to a MGUS, chemotherapy directly against the pathological clone was preferred [30]. Recent evidences from several observational studies has further supported a clone-directed approach to treat C3G with monoclonal gammopathy [12, 16]. In the study of Chauvet et al., treatment with clone-directed chemotherapy (29 patients, including bortezomib in 22 patients), compared with treatment without chemotherapy (21 patients, including either immunosuppressive therapy or conservative therapy), was associated with an improved rate of renal response and improved renal survival [12]. Importantly, renal survival was significantly increased among patients achieving a homological response. Data from the Mayo Clinic showed that targeted therapy against the monoclonal gammopathy in 16 patients (five of 16 had MGRS; others had more advanced hematological disease) resulted in a complete or partial renal response or stable renal function in 44% of patients. Furthermore, 17 patients (all of them had MGRS) received non-targeted therapy (mostly glucocorticoids alone or in combination with other immunosuppressive agents), and non-targeted therapy resulted in a complete or partial renal response in 41% of patients [16].

Herein, we reported a patient with C3GN who had anti-CFH IgG activity and a monoclonal gammopathy. We further proved that the functional autoantibody was borne by the monoclonal IgGλ, and it is the first time that intact monoclonal immunoglobulin could act as an anti-CFH antibody and lead to MGRS-associated C3GN by activating the CAP.

## Data Availability

The data used and analyzed during the current study are available from the corresponding author on reasonable request.

## Abbreviations

aHUS: Atypical haemolytic uremic syndrome
AMD: Age-related macular degeneration
C3G: C3 glomerulopathy
C3GN: C3 glomerulonephritis
C3NeF: C3 nephritic factor
CAP: Complement alternative pathway
CFH: Complement factor H
COS-P: C3NeF stabilization ELISA with properdin
DDD: Dense deposit disease
GBM: Glomerular basement membrane
MGRS: Monoclonal gammopathy of renal significance
MGUS: Monoclonal gammopathy of undetermined significance
MIg: Monoclonal immunoglobulins
